# Steer’n’Detect: fast 2D template detection with accurate orientation estimation

**DOI:** 10.1093/bioinformatics/btac270

**Published:** 2022-04-18

**Authors:** Virginie Uhlmann, Zsuzsanna Püspöki, Adrien Depeursinge, Michael Unser, Daniel Sage, Julien Fageot

**Affiliations:** European Bioinformatics Institute, European Molecular Biology Laboratory (EMBL), Cambridge, UK; School of Engineering, Biomedical Imaging Group, Ecole Polytechnique Fédérale de Lausanne (EPFL), Lausanne, Switzerland; Institute of Information Systems, University of Applied Sciences Western Switzerland (HES-SO), Sierre, Switzerland; School of Engineering, Biomedical Imaging Group, Ecole Polytechnique Fédérale de Lausanne (EPFL), Lausanne, Switzerland; School of Engineering, Biomedical Imaging Group, Ecole Polytechnique Fédérale de Lausanne (EPFL), Lausanne, Switzerland; School of Computer and Communication Sciences, AudioVisual Communications Laboratory, Ecole Polytechnique Fédérale de Lausanne (EPFL), Lausanne, Switzerland

## Abstract

**Motivation:**

Rotated template matching is an efficient and versatile algorithm to analyze microscopy images, as it automates the detection of stereotypical structures, such as organelles that can appear at any orientation. Its performance however quickly degrades in noisy image data.

**Results:**

We introduce Steer’n’Detect, an ImageJ plugin implementing a recently published algorithm to detect patterns of interest at any orientation with high accuracy from a single template in 2D images. Steer’n’Detect provides a faster and more robust substitute to template matching. By adapting to the statistics of the image background, it guarantees accurate results even in the presence of noise. The plugin comes with an intuitive user interface facilitating results analysis and further post-processing.

**Availability and implementation:**

https://github.com/Biomedical-Imaging-Group/Steer-n-Detect.

**Supplementary information:**

Supplementary data are available at *Bioinformatics* online.

## 1 Introduction

Rotated template matching is commonly used when analyzing microscopy images of biological structures as it allows automating the detection of objects of interest, such as organelles, that may appear at any random orientation (Lucas *et al.*, 2021). This approach requires a single training example, giving it a strong edge over machine learning-based methods whenever objects of interest vary little in appearance. Template matching performance, however, degrades in the presence of background noise, resulting in erroneous and missed detections. Here, we present Steer’n’Detect, an ImageJ plugin implementing our recently published algorithm (Fageot *et al.*, 2021) for fast template detection at any orientation in 2D images, relying on steerable filters. Steer’n’Detect is more robust to noise and faster than rotated template matching, while remaining equivalently accurate in its orientation estimation. As such, Steer’n’Detect can reliably be used in 2D microscopy images with high levels of background noise, where template matching would perform poorly.

## 2 Approach and implementation

The principle of the algorithm implemented in Steer’n’Detect is explained in Fageot *et al.* (2021). We hereafter recall the main ideas of this approach.

We model the input image as several copies of a template *T* occurring at various and unknown positions and orientations in some additive background noise *S*. Our method aims at recovering these unknown positions and orientations. The background noise *S* is assumed to be a 2D self-similar isotropic Gaussian random field, which is an appropriate model for microscopy images (Sage *et al.*, 2005). The power spectrum of *S* is then given by $\sigma^{2}\omega^{\gamma }$, where the variance $\sigma^{2}>0$ reflects the noise intensity and the exponent γ≥0 characterizes its smoothness (higher values result in smoother noise). A detector can efficiently retrieve occurrences of *T* in the image if it responds strongly to the template while being maximally insensitive to the background noise *S*. Since we assume the template of interest *T* to be present in the image at an arbitrary unknown orientation, the detector must also be able to rotate at low computational cost. Taking this into account, the algorithm implemented in Steer’n’Detect designs steerable filters that achieve optimal detection performance while minimizing response to noise in a process called spectral shaping. Steerable filters have been used for the detection of symmetrical and directional structures in biological images (Püspöki *et al.*, 2016) and were recently demonstrated to be powerful tools to design detectors based on a template (Zhao and Blu, 2020). They can efficiently approximate any template up to a user-defined accuracy and can be continuously rotated at any arbitrary angle, resulting in computationally light detection pipelines.

**Fig. 1.**
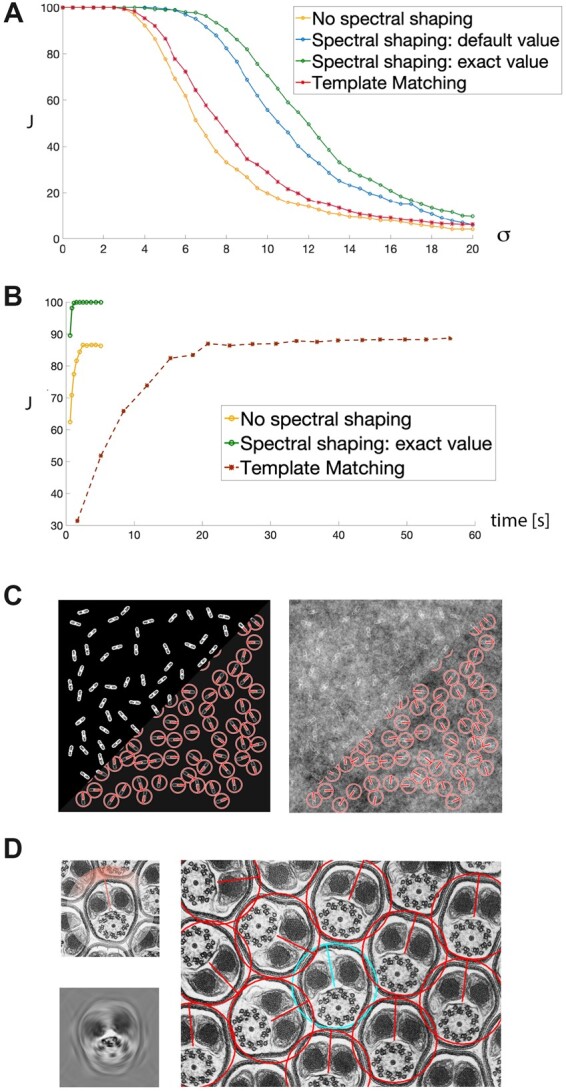
(**A**) Performance comparison of template matching, adapted steerable filters (no spectral shaping), and Steer’n’Detect with *γ* = 1 (optimal) and γ=0.5 (suboptimal), measured by the Jaccard index *J* as a function of the amount of background noise (standard deviation *σ*). (**B**) Performance comparison of rotated template matching, adapted steerable filters (no spectral shaping) and Steer’n’Detect, measured by the Jaccard index *J* as a function of run time, for a fixed background signal (*σ* = 5). (**C**) Detection of rod-shaped bacteria with Steer’n’Detect (synthetic image degraded by self-similar Gaussian random field background noise). (**D**) Detection of spermatozoan axoneme with Steer’n’Detect (transmission electron microscopy image) (Reused from http://www.cellimagelibrary.org/images/35970). Top left: image crop used as template; bottom left: resulting detector; right: detection results

In Steer’n’Detect, the implementation of this approach involves of the following steps:

1. **Detector design**: The steerable detector is automatically constructed from a single template *T* provided as input. Spectral shaping is performed by tuning a self-similarity parameter *γ* to the image background *S*. Technical details, including the full derivation of the detector, are provided in (Fageot *et al.*, 2021). The value of *γ* can be estimated by different procedures, but getting it exactly right is not critical as good results can be obtained even for imprecise guesses (see Fig. 1A).
2. **Image processing**: The orientation resulting in the strongest detector’s response, as well as the magnitude of the response itself, are computed for each pixel of the input image through an optimization process. At any location in the image, the output reflects to which extent the pattern of interest is present (through the magnitude of the detector’s response) and how it is oriented. Local non-maximum suppression is applied on the detector’s magnitude response map to prevent repeated detection of the same template occurrence.
3. **Selection** **and** **visualization**: Detection results meeting a user-defined quality threshold based on the magnitude of the detector’s response can be explored, visualized and exported for further processing.

## 3 Results and validation

Steer’n’Detect is best applicable to detect occurrences of a template repeated in 2D images at arbitrary location and orientation, and possibly buried in background noise. Steer’n’Detect can thus be used as a faster alternative to rotated template matching, and produce accurate results when the latter fails due to noise. To demonstrate this, we produced 480 × 480 synthetic images containing 100 occurrences of simulated rod-shaped structures akin to *Escherichia* *coli* bacteria or *Schizosaccharomyces* *pombe* yeasts. The position of the objects to be detected and their orientations are known as ground-truth. We then degrade these synthetic data with background noise based on the Gaussian random field introduced in Section 2. Using a linear assignment algorithm to pair the detections with ground-truth locations, we consider a detection to be a correct if its distance to the closest template occurrence is within a 5 pixels range. We then use the Jaccard index to quantify the overall detection performance and report it (in percentage) as a function of the amount of background noise in Figure 1A, and as a function of algorithmic run time in Figure 1B. Our experiment shows that, in the presence of background noise, the Steer’n’Detect provides better results than both template matching and adapted steerable filters, even when the self-similarity parameter is left to its default value. Template matching swept through all orientations slightly outperforms classical steerable filters (no spectral shaping) in low noise regime, since they only provide a rough approximation of the template. However, computation time is an order of magnitude longer for template matching than for any considered approach.

In addition to detection performance, we also quantitatively compare the angular accuracy as a function of run time. To be able to focus solely on orientation estimation, we generate synthetic images containing a single rod-shaped structure at their center, rotated at a random angle. We then corrupt these images with background noise as previously. For each algorithm, we report the root mean square (RMS) difference between the retrieved orientation estimates and their corresponding ground truth values after 60 s of run time. We obtain an RMS difference of 0.515° for template matching and 0.343° for Steer’n’Detect. A comprehensive performance assessment and validation of the algorithm can be found in Fageot *et al.* (2021).

Finally, in Figure 1D, we illustrate Steer’n’Detect results on real biological data: our method is able to accurately detect axoneme (and their orientation) on a transmission electron microscopy image of a *Culex* mosquito spermatozoa flagellum (Cell Image Library, CIL:35970).

## Data availability

The data underlying this article are available at http://bigwww.epfl.ch/algorithms/steer_n_detect/ and were partially derived from CC-BY sources from the Cell Image Library (cil:35970).

## Funding

This work was supported by EMBL internal funding (V.U.) and the Swiss National Science Foundation (SNSF) [P400P2_194364 to J.F., 200020_184646/1 to M.U., 205320_179069 to A.D.].

*Conflict of Interest*: none declared.

## Supplementary Material

btac270_Supplementary_Data
