# Supplementary material for: Steer’n’Detect: fast 2D template detection with accurate orientation estimation
Source: Bioinformatics. 2022 Apr 18;38(11):3146–8. doi: 10.1093/bioinformatics/btac270 (PMC9154219; doi:10.1093/bioinformatics/btac270)
Supplement: btac270_Supplementary_Data [file btac270_supplementary_data.pdf]

Bioinformatics
doi.10.1093/bioinformatics/xxxxxx
Advance Access Publication Date: Day Month Year
Manuscript Category

OXFORD

Supplementary Material

Steer'n'Detect: Fast 2D Template Detection with Accurate Orientation Estimation

Virginie Uhlmann [1,*,†], Zsuzsanna Püspöki [2,†], Adrien Depeursinge [3], Michael Unser [2], Daniel Sage [2], and Julien Fageot [4]

[1] European Bioinformatics Institute, European Molecular Biology Laboratory (EMBL), Cambridge, UK.
[2] Biomedical Imaging Group, Ecole polytechnique fédérale de Lausanne (EPFL), Lausanne, Switzerland.
[3] Institute of Information Systems, University of Applied Sciences Western Switzerland (HES-SO), Sierre, Switzerland.
[4] AudioVisual Communications Laboratory, Ecole polytechnique fédérale de Lausanne (EPFL), Lausanne, Switzerland.

[*] To whom correspondence should be addressed.
[†] These authors contributed equally to this work.

Associate Editor: XXXXXXX

Received on XXXXX; revised on XXXXX; accepted on XXXXX

Abstract

This document contains supplementary material to the Steer'n'Detect manuscript.

1 Steer'n'Detect at a Glance

The main features of the ImageJ plug-in are as follows.

- Steer'n'Detect automatically retrieves the locations and orientations of occurrences of a user-provided template across an input image;
- The resulting detections are given an unique ID and returned in a table displaying their location (x and y image coordinates), angle, confidence, and type. Steer'n'Detect uses adapted steerable filters to detect occurrences of the template. The strength of the filter's response can thus be used as a proxy for detection certainty and is the value reported in the table as measure of confidence. Detections can either be retrieved automatically by the algorithm or added/edited manually by the user. This is reflected by the detection type, labelled either as "auto" or "manual + timestamp". Note that the orientation of the detection is always determined automatically.
- The detections are displayed as circles with an inscribed segment indicating their estimated orientation. Detections can in addition be labelled either by their ID, orientation, or confidence level. Their centers can optionally be indicated as a cross.
- The detection table and the visualization are interactively linked. When a row is selected in the table, the corresponding detection will be highlighted in the image in a different color. Conversely, if a detection is selected in the image, then the corresponding row will be highlighted in the table.
- All data contained in the detection table can be further processed (see below) or exported as a comma-separated values (CSV) file.

The available post-processing actions are as follows:

- Select detections within a range of angular accuracy (angle), location (coordinates in x and y), or confidence.
- Manually add further detections at a user defined position (double-click on the image). The orientation of the detection is then automatically computed by Steer'n'Detect.
- Manually remove detections (select detection on the image and hit "delete").
- Manually reposition detections (click and drag). When manually repositioning a detection, Steer'n'Detect computes the detector's response and optimal orientation on the fly and automatically updates the detection circle, providing immediate visual feedback.
- Save results as a CSV file or load previous measurements.

2 User interfaces and modes

The software has two user interfaces: regular (Steer'n'Detect) and load (Steer'n'Detect Load).

2.1 Steer'n'Detect

The Steer'n'Detect user interface compute detection results on an input image and collect them in the detection table. When computations are finished, the software switches to post-processing mode, enabling the user to edit the results and export them.

© The Author 2021. Published by Oxford University Press. All rights reserved. For permissions, please e-mail: journals.permissions@oup.com

2.2 Steer’n’Detect Load

The Steer’n’Detect Load user interface loads previously saved measurements (stored as a CSV file) directly to the Steer’n’Detect interface. This allows directly visualizing and further editing previously saved results. The “Browse” button allows the user to set the path to the file that is to be loaded.

3 General Information

3.1 Pre-requirements

Steer’n’Detect is a plugin for ImageJ, a general purpose image-processing and image-analysis software (Schneider *et al.*, 2012). ImageJ has a public domain license (GNU GPL-3.0) and runs on most operating systems. It does not take more than a couple of minutes to install. Steer’n’Detect was developed on ImageJ version 1.53s and Java 1.8.0.

The user is required to have a recent versions of Java, we recommend at least version 1.8. For processing large data sets, it is preferable to have a 64-bit version. The Help menu (Help → About) of ImageJ provides information about the current version of Java.

3.2 Download and Install

The Steer’n’Detect plugin can be downloaded from the website `bigwww.epfl.ch/algorithms/steer_n_detect`. Once downloaded, it must be placed in the plugins folder of the ImageJ directory. No external libraries are required by the plugin. There is a possibility that the Java allocated memory of ImageJ is not sufficient. In that case, memory can be manually increased (Edit → Options → Memory and Threads), though it is not recommended to set it above $3/4$ of the available physical memory of the machine.

3.3 Conditions of Use

The software is distributed under GNU GPL-3.0 License. We expect users to include a citation of this publication whenever presenting or publishing results that are based on the ImageJ plugin Steer’n’Detect.

3.4 Reporting a Bug

We recommend using the latest version of the Steer’n’Detect software. Before submitting a bug report, the user should make sure that the issue has not been corrected by the latest upgrade. In case of a malfunction, the user can contact the authors at one of the e-mail addresses provided on the plugin website.

When submitting a bug report, we ask to specify the malfunction and describe the procedure to reproduce the error. The description should contain the corresponding platform details, including the version of Java, ImageJ, Steer’n’Detect, and the operating system.

3.5 Additional Instructions

- The plugin requires a grayscale image at launch.
- The input image file can be i) dragged and dropped from the operating system’s file explorer to ImageJ, or ii) opened by selecting File → Open in ImageJ and browsing.
- Steer’n’Detect supports the following image formats: 8-bit, 16-bit and 32-bit. In the case of a stack of images, processing is always done on the currently visible image slice.
- Steer’n’Detect is “attached” to the image that is being processed. Once a new image is opened, a new instance of Steer‘n’Detect must be opened to consider the new image as input.
- The Steer’n’Detect plugin can be recorded as an ImageJ macro and used further for batch processing.

4 Steer’n’Detect Interface

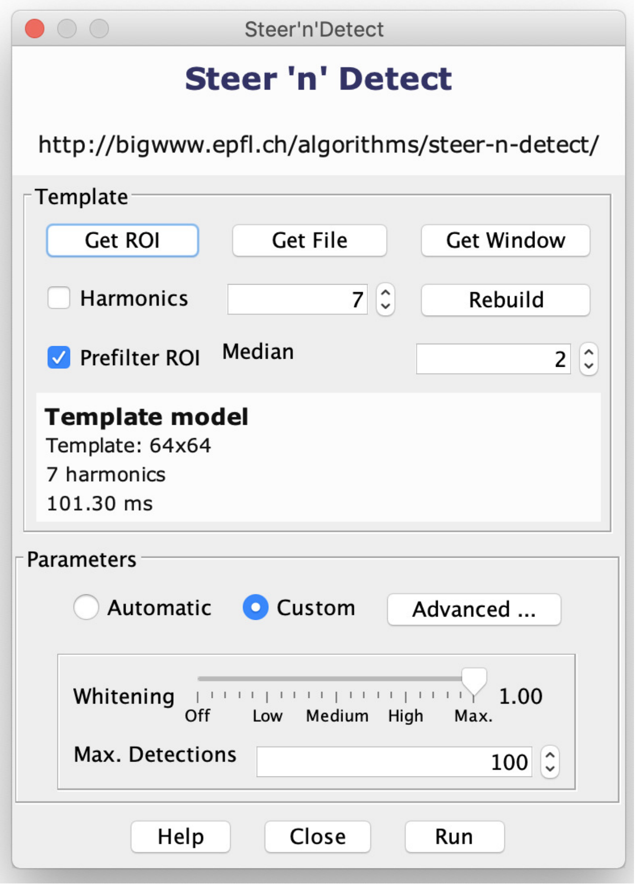

Fig. 1. Interface of the Steer’n’Detect plugin at launch.

4.1 Template

There are three possible ways to provide a template.

- **Get ROI**: A region of interest can be selected on the image by placing the circular cursor of Steer’n’Detect on the image and then clicking on the ”Get ROI” button. The width of the template and its reference orientation can be manually adjusted.
- **Get File**: A template image can be loaded by clicking on ”Get File”,
- **Get Window**: One of the open image windows of ImageJ can be used as a template image by clicking on ”Get Window”.

Once the template is chosen, its resulting steerable filter representation is displayed in a new window. The number of **Harmonics** can be adapted to provide a finer or coarser approximation of the template. In general, the higher the number of harmonics, the better the approximation. Increasing the number of harmonics increases the run time and makes the results more sensitive to noise. However, a good detection rate can already be achieved with a small number of harmonics. When changing the number of harmonics, the template approximation has to be recomputed; hence pressing the **Rebuild** button is necessary.

Prefilter ROI: Pre-filtering the template can improve the detection in the presence of strong image background noise. In the current implementation, we are using a median filter, where each pixel of the template is replaced with the median of its neighbors. The median parameter corresponds to the size of the window/radius when choosing the neighbors.

Information Window: The Information Window provides information on the template that is used for detection.

4.2 Parameters

Processing parameters are set automatically based on the properties of the input image (*e.g.*, its size), but can also be adjusted manually.

The Advanced Parameters Settings Box contains a set of parameters that the user can adjust before processing the data.

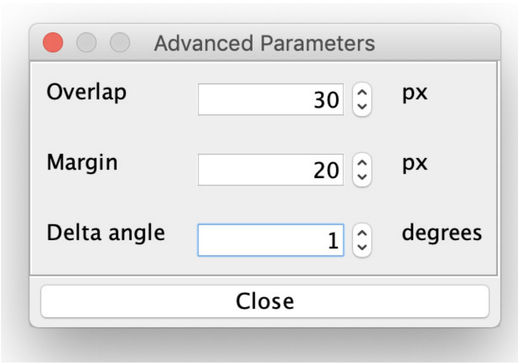

Fig. 2. The parameter setting box.

- **Overlap**: The maximal size of the overlap (in pixels) that is allowed for intersecting detections. The default value is 0, which means that no overlap is allowed between detections.
- **Margin**: The user can narrow down the search space of the detections by excluding regions close to the boundaries of the image. The margin size is set in pixels.
- **Delta angle**: The precision of the orientation estimate (the bigger the delta, the rougher the estimation). Set in degrees.

Spectral shaping: Spectral shaping greatly improve detection performance in the presence of noise. The spectral shaping parameter quantifies the smoothness of the background from rough (shaping 0) to smooth (shaping 1). For further information on the spectral shaping process, we refer to Fageot *et al.* (2021).

Max. Detections: The maximum number of objects that the user intends to detect. It is considered as a theoretical upper bound, and the number of detections might be smaller than this limit.

5 Results Panel

Once the detection phase is complete, the user interface automatically switches to the results panel (Figure 3) and displays the detections overlaid on the input image. Initially, all detections are visualized and collected into a table.

5.1 Selection Tab

The following checkboxes can be found in the selection tab of the results panel.

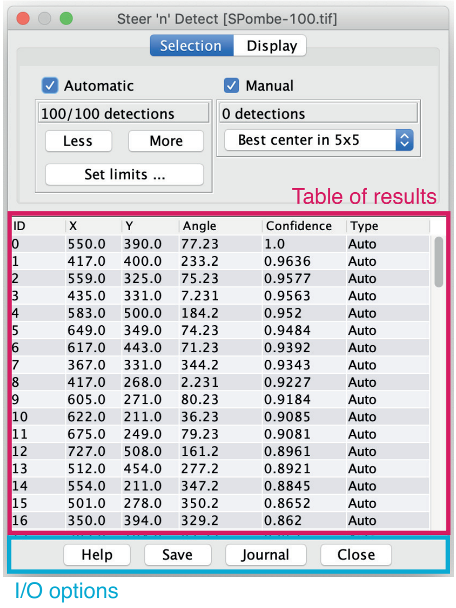

Fig. 3. The results panel, featuring selection and display tabs, a table of results, and input/output (I/O) options.

- **Automatic**: Visualizes (in the table and on the image) the detections made by the software.
- **Manual**: Visualizes (in the table and on the image) the detections that were added manually (if any).

The following keys can be found in the selection tab of the results panel..

- **Less**: This key allows removing detections based on their confidence. Only automatically calculated detections are removed.
- **More**: This key allows adding further detections based on their confidence.

5.1.1 Detection Selection - Set limits

One may wish to select detections based on some criteria. To set the parameters for selection, one must click on the **Set limits...** key in the selection tab. The table of results is automatically sorted according to the chosen measure.

- **X Lower/Upper Limit**: Selects only detections whose estimated positions, with respect to the x coordinate (horizontally), fall in the indicated range.
- **Y Lower/Upper Limit**: Selects only detections whose estimated positions, with respect to the y coordinate (vertically), fall in the indicated range.
- **Angle Lower/Upper Limit**: Selects only detections whose estimated orientation fall in the indicated range.
- **Confidence Lower/Upper Limit**: Selects only detections whose confidence level falls in the indicated range. Depending on the application, there are two possible approaches to treat the number of false positive/negative results. In some cases, one might prefer to consider all detections, accepting some false ones; in other instances, it may be favorable to keep fewer detections with a high level of confidence. The confidence level measure addresses this by enabling the user to filter out the least reliable detections. Higher confidence level corresponds to a higher level of certainty that the detections are correct.

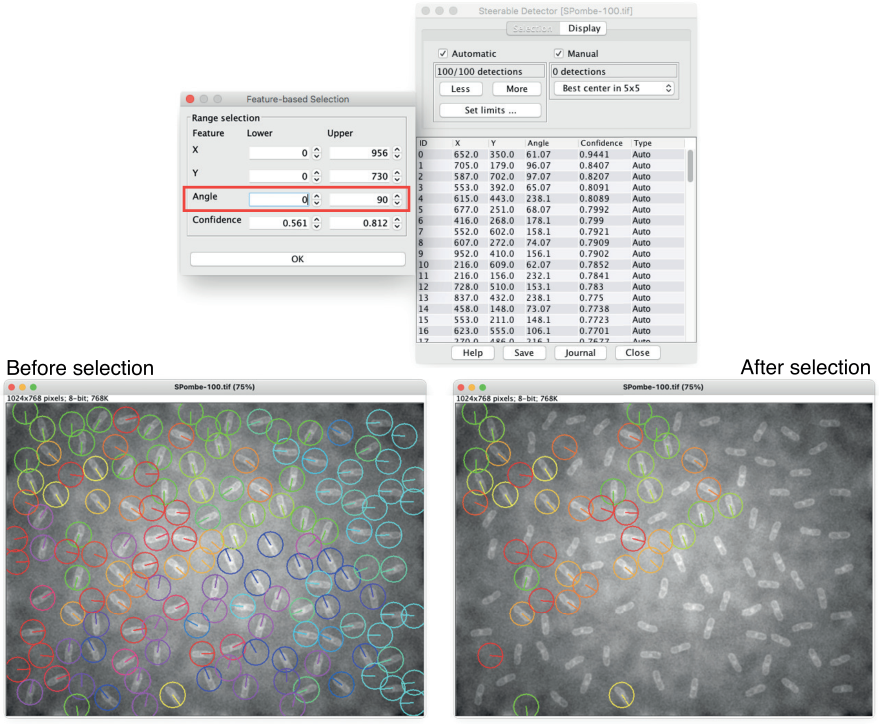

Fig. 4. Sorting results with set limits. In this example, all detections are initially color-coded according to their orientation (left), and results with an angle falling between 0 and 90 degrees to the original template orientation are selected (right).

Note that the selection will only apply to the results currently in the table. If new results are manually added or existing results modified (see Section 6), **Set limits...** should be used again to sort this new collection of results.

5.1.2 Optimization of the Center

The option “Best center in $M \times M$” helps the user when manually editing the results. When adding or dragging a detection, Steer’n’Detect automatically searches for the highest detection response in an $M \times M$ window around the mouse cursor. It means that there is no need to exactly click to the center point of the detection. Instead, Steer’n’Detect finds it automatically. The size of the search window can be adjusted by the user. The following settings are allowed: either no optimization of the center, or optimization of the center in an $M \times M$ window, where M equals to 3, 5, 7, 9, 11, or 13.

5.2 Display Tab

The display tab (Figure 5) allows adjusting the visualization of the results with the following options.

- **Show Outer**: Specifies the color used to visualize the detections. The corresponding number denotes the thickness of the stroke.
- **Show Inner**: The corresponding number denotes the opacity of the circle, higher numbers correspond to less transparency.
- **Show Label**: The possible choices are: no label, ID, angle, confidence. One can specify the color of the label.
- **Show center:** The center of the detections is marked with a cross. One can specify its color.
- **Screenshot:** Saves a picture of the detections overlaid on top of the input image. This screenshot can be saved into any image format supported by ImageJ.

5.3 Table of Results

The table of results (see Figure 3) contains all the information gathered about detections, and allows the user to easily interact with them.

- Information provided by the table of results: detection ID, coordinates of the detection (horizontal x and vertical y), orientation, corresponding confidence level, and type of the detection.
- The table of results is originally ordered based on confidence level. The ordering can be done based on the other values (*e.g.*, detection ID, coordinates) by clicking on the top of the corresponding column.
- Each detection has its own unique identification number.
- The detection type can be either “Auto” or “Manual + timestamp”. The orientation of the detection is always determined automatically.
- Once a detection row is highlighted in the table, the corresponding detection is also highlighted in the image overlay. Conversely, once a detection is highlighted in the image (by clicking on it), the corresponding row becomes highlighted in the table.

For more information on how to interpret the confidence level and for a quantitative evaluation of the accuracy of the orientation estimation, we refer the reader to Fageot *et al.* (2021).

5.4 Input/Output

The input/output options can be found at the bottom of the Results Panel (see Figure 3).

- **Help**: Directs the user to the website of the plugin. There, one can find further information and download the latest version of the plugin.
- **Save**: Saves the detections as a CSV file so that they can be be further processed later on.
- **Journal**: Summarizes the most important details about the execution of the plugin (*e.g.*, settings, run time, memory allocations).

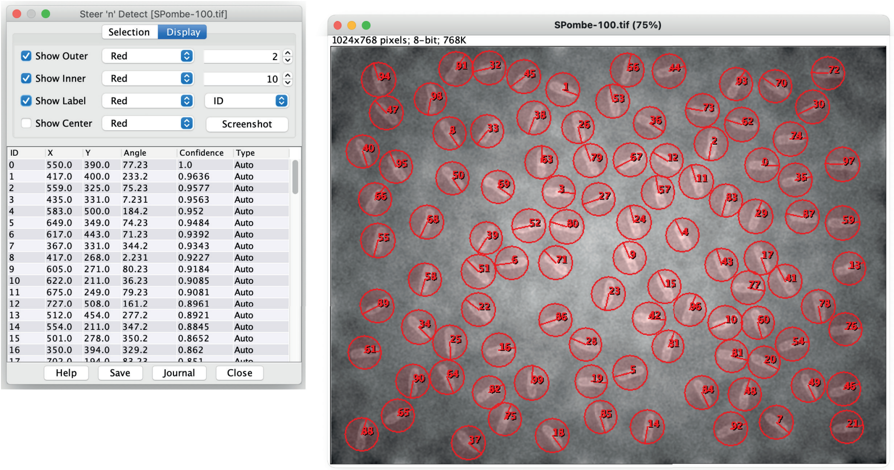

Fig. 5. The display panel, with an example of results vizualized with red outer strokes, transparent red inner fill, and labels.

- **Close**: Closes the plugin and saves current parameters. Note that there is a difference between closing the plugin by clicking on the close key and simply closing the window, since parameters are not saved in the latter case.

6 Interacting with Detections

It is possible to interact with the detections directly.

- **Adding a detection**: The user must double-click on the location of the center of the detection to be added. A new detection will appear in the detection list with an automatically computed orientation. The type of the detection in the table of results reflects the fact that it was added by the user by being denoted as "Manual + timestamp".
- **Removing a detection**: To remove a detection, the user must select it first and then press the "delete" key. The previously detected object will disappear from the image and from the table of results.
- **Click and drag option** : The click and drag option facilitates the process of adding new detections. A detection can be dragged around, holding the mouse button down, until the user is happy with its location. The orientation of the detection is computed automatically. The corresponding entry in the table of results is refreshed on the fly. The values of the detection's X and Y position, confidence, and angle are therefore interactively updated as the user drags the detection.

Interacting with detections will modify the results table. As such, any results filter introduced in Section 5.1.1 should be reapplied to sort the new collection of results.

Funding

The authors are supported by EMBL internal funding (VU) and by the Swiss National Science Foundation (SNSF) under grants P400P2_194364 (JF), 200020_184646/1 (MU) and 205320_179069 (AD).

References

Fageot, J. *et al.* (2021). Principled design and implementation of steerable detectors. *IEEE Transactions on Image Processing*, **30**, 4465–4478.

Schneider, C. *et al.* (2012). NIH Image to ImageJ: 25 years of image analysis. *Nature Methods*, **9**(7), 671–675.
